# Phrenology: A Protest

**Published:** 1888-07-07

**Authors:** Chas. E. Holland


### THE EDITOR'S LETTER-BOX.

[Our Correspondents are reminded that prolixity is a great bar to publication, and that brevity of style and conciseness of statement greatly facilitate early insertion.]

### PHRENOLOGY.—A PROTEST.

Taking advantage of your column for expressing "Everybody's Opinion," I think that any contributor to a scientific journal ought, in the interests of science, to state facts. The pages which it chooses to devote to scientific information should not be made the vantage-ground of mental speculation or whimsical suppositions. Several arrows have been previously shot at phrenology by your contributors, but the climax of injustice in respect to that science appears to have been reached by a writer in last week's issue of The Hospital. In an article entitled the "Human Skull" we learn the writer's definition of phrenology. His definition is not a good one. He commences by saying that "the so-called science of phrenology is a theoretical subject." It may prove of service to the truth-seeker to observe here that phrenology, so far as legal and medical evidence goes, was proved and accepted as a "science" many years ago. That phrenology has a theoretical side is quite true; that phrenologists rely upon the theoretical side is positively untrue. Dr. L. N. Fowler, an eminent phrenologist, says in one of his works that "theoretical phrenology, like speculative metaphysics, is valueless. . . . And it is to experimental tribunal alone that phrenology makes her appeal." The writer goes on to say that phrenology is discredited among physiologists. Is he prepared to say that Mr. Abernethy, Mr. Lawrence, Dr. Samuel Solly, Dr. Mayo, Dr. Todd, Dr. Lardner, Dr. Laycock, Dr. Noble, Professor Hunter, M.D., are not physiologists? He must either prove so or else believe phrenology to be true; for these authorities, which are still held in the highest respect by the medical world, have each, with many others, given their unqualified support to phrenology, and many of them regard with great honour its founders, Drs. Gall and Spurzheim. He continues, "phrenology, therefore, as phrenologists understand it—that is, the mapping out of a man's character by means of the bumps and hollows of his head—is a perfectly impossible science." What possibilities of bumpology may be I know not, but this I know, that phrenology is not dependent in the slightest upon the "bumps and hollows of the head." Many heads, indeed, have none; yet their phrenology is manifest. For your correspondent to say positively that his definition of the science is that of its practitioners, says, in my humble opinion, a great deal for his presumption. I venture to say that he cannot mention any text-book on phrenology—and there are many—that would justify such an eccentric interpretation; well may he say, upon a bumpological foundation, that it is a perfectly impossible science. He continues: "There is, however, a true brain science." If this is so, what is it called? Surely a science of such importance should possess a name. That "different parts of the brain have different functions" was known to phrenologists and others before Dr. Ferrier was. That "Ferrier and other physiologists have succeeded in localising many of the functions with a good deal of precision," is simply a corroboration of phrenological truths, for Dr. Ferrier himself says, "So far, the facts of experiment and disease favour the view of the phrenologist." This is saying a great deal more for phrenology than your correspondent finds himself able to say, and issues from the very source whence he considers the science to be over-ridden. It is conclusive that while one man has written what he knew, the other has written what he merely supposed. Can the information, however, afforded by Dr. Ferrier's experiments be so reliable? I do not think myself that one can place reliance upon data furnished in trying to ascertain natural phenomena by unnatural means. The scalpel, hot iron, and the electric rod will never produce by their action upon the brain a similarity of action to the mind, notwithstanding the fact of your correspondent's remark that these experimentalists localise function with "a good deal of precision."

Sir, I will trespass but little further upon your valuable space. There is just one other statement which, in the interests of truth, I think it needful to note. The writer says "the excess or defect of any intellectual or moral faculty certainly cannot be predicted from the outward conformation of the head." Dr. Johnson, however, editor of the "Medical and Chirurgical Review," says: "Those who sneer at phrenology are neither anatomists nor physiologists. Special mental qualities have a special conformation of the head." Probably Dr. Johnson understands phrenology as phrenologists understand it.

Trusting, on behalf of a cause alike truthful and useful, that that you will insert this, I am, etc.,

Chas. E. Holland.

P.S.—A non-professional phrenologist.

